# Mouse-adapted SARS-CoV-2 protects animals from lethal SARS-CoV challenge

**DOI:** 10.1371/journal.pbio.3001284

**Published:** 2021-11-04

**Authors:** Antonio Muruato, Michelle N. Vu, Bryan A. Johnson, Meredith E. Davis-Gardner, Abigail Vanderheiden, Kumari Lokugamage, Craig Schindewolf, Patricia A. Crocquet-Valdes, Rose M. Langsjoen, Jessica A. Plante, Kenneth S. Plante, Scott C. Weaver, Kari Debbink, Andrew L. Routh, David Walker, Mehul S. Suthar, Pei-Yong Shi, Xuping Xie, Vineet D. Menachery

**Affiliations:** 1 Departments of Biochemistry and Molecular Biology, University of Texas Medical Branch, Galveston, Texas, United States of America; 2 Departments of Microbiology and Immunology, University of Texas Medical Branch, Galveston, Texas, United States of America; 3 Department of Pediatrics, Emory Vaccine Center, Emory University School of Medicine, Atlanta, Georgia, United States of America; 4 Department of Pathology, University of Texas Medical Branch, Galveston, Texas, United States of America; 5 World Reference Center of Emerging Viruses and Arboviruses, University of Texas Medical Branch, Galveston, Texas, United States of America; 6 Institute for Human Infection and Immunity, University of Texas Medical Branch, Galveston, Texas, United States of America; 7 Department of Natural Science, Bowie State University, Bowie, Maryland, United States of America; 8 Yerkes National Primate Research Center, Atlanta, Georgia, United States of America; New York University School of Medicine, UNITED STATES

## Abstract

The emergence of Severe Acute Respiratory Syndrome Coronavirus 2 (SARS-CoV-2) has resulted in a pandemic causing significant damage to public health and the economy. Efforts to understand the mechanisms of Coronavirus Disease 2019 (COVID-19) have been hampered by the lack of robust mouse models. To overcome this barrier, we used a reverse genetic system to generate a mouse-adapted strain of SARS-CoV-2. Incorporating key mutations found in SARS-CoV-2 variants, this model recapitulates critical elements of human infection including viral replication in the lung, immune cell infiltration, and significant in vivo disease. Importantly, mouse adaptation of SARS-CoV-2 does not impair replication in human airway cells and maintains antigenicity similar to human SARS-CoV-2 strains. Coupled with the incorporation of mutations found in variants of concern, CMA3p20 offers several advantages over other mouse-adapted SARS-CoV-2 strains. Using this model, we demonstrate that SARS-CoV-2–infected mice are protected from lethal challenge with the original Severe Acute Respiratory Syndrome Coronavirus (SARS-CoV), suggesting immunity from heterologous Coronavirus (CoV) strains. Together, the results highlight the use of this mouse model for further study of SARS-CoV-2 infection and disease.

## Abstract 2

Studying cross-protection from different coronaviruses is important to inform the research for a universal vaccine. This study uses a mouse-adapted SARS-CoV-2 strain to show that it confers protection from SARS-CoV challenge, suggesting possible immunity from heterologous challenge following natural infection.

## Introduction

Severe Acute Respiratory Syndrome Coronavirus 2 (SARS-CoV-2), the virus that causes Coronavirus Disease 2019 (COVID-19), emerged in late 2019 and has since caused an ongoing pandemic with over 153 million cases and over 3.2 million deaths in the last 17 months [1,2]. The novel coronavirus, similar to previous emergent Severe Acute Respiratory Syndrome Coronavirus (SARS-CoV) and Middle East Respiratory Syndrome Coronavirus (MERS-CoV), can produce severe respiratory disease characterized by fever, labored breathing, and pulmonary infiltration and inflammation [3,4]. In severe cases, SARS-CoV-2 can lead to acute respiratory distress and death. Unlike the earlier pandemic Coronaviruses (CoVs), SARS-CoV-2 maintains the ability to efficiently spread asymptomatically and causes a range of disease from mild to severe [5]. These factors have led to a pandemic that continues to rage over a year after its emergence.

In responding to the outbreak, understanding the complexity of SARS-CoV-2 infection has been hampered by the limitations of small animal models [6]. Early on, wild-type (WT) SARS-CoV-2 was shown to be unable to use mouse angiotensin converting enzyme 2 (ACE2) for entry and infection [7]. Alternative models use receptor transgenic mice expressing human ACE2 or Syrian golden hamsters to evaluate SARS-CoV-2 infection and disease in vivo [6]. However, the transgenic models, while causing severe disease and lethality, have distinct infection tropism, leading to encephalitis in addition to lung disease [8–10]. Similarly, while the hamster model has provided use in studying disease and transmission [11], the absence of genetic knockout and immunological tools limits the types of studies that can be pursued. Without a robust mouse model, many of the resources used to study infection and the immune response are unavailable for SARS-CoV-2 experiments.

In order to alleviate these issues, we set out to develop a mouse-adapted strain of SARS-CoV-2 using standard laboratory strains. Building from our infectious clone system [12], we incorporated amino acid changes that facilitated replication in standard BALB/c mice and serially passaged the mutant to create a mouse-adapted strain (CMA3p20) that causes significant weight loss, disease, and lung damage following infection. Notably, virus replication in this model is limited to the respiratory system, thus recapitulating disease observed in most humans. Importantly, the SARS-CoV-2 CMA3p20 strain did not attenuate replication in primary human airway cultures or change the antigenicity of the mouse-adapted strain relative to WT control virus, making it suitable for vaccine and therapeutic studies. Finally, following prior infection with SARS-CoV-2 CMA3p20, mice were protected from lethal challenge with SARS-CoV despite the absence of sterilizing immunity. Adoptive serum transfer experiments indicated that protection was not mediated by antibody alone. Together, the results highlight the use of SARS-CoV-2 CMA3p20 to study infection and pathogenesis in standard mouse lines.

## Results

The initial, emergent strains of SARS-CoV-2 had spike proteins unable to utilize mouse ACE2 and infect standard laboratory mice [7]. To overcome this barrier, we generated a series of mutations in the receptor-binding domain (RBD) of SARS-CoV-2 using our infectious clone [12]. Our initial efforts modeled the interaction between SARS-CoV-2 and mouse ACE2 and used previous mouse-adapted strains of SARS-CoV (MA15, MA20, and v2163) [13] to design mutants including changes at Y449H (MA1), Y449H/L455F (MA2), and F486L/Q498Y (MA4) (**S1A–S1C Fig**). We also generated a series of mutants based on a reported natural SARS-CoV-2 isolate (MASCP6) capable of infecting mice [14], which has spike change at N501Y and several additional mutations (**S2A Fig**). Given the capacity of the MASCP6 strain to replicate in mice, we generated mutants that had the spike mutation alone (CMA1), the spike/N protein mutation (CMA2), and all 4 changes (CMA3) (**S2A Fig**). For each of the 6 mutants, we used site-directed mutagenesis in the WA1 strain clone and rescued virus stocks on Vero E6 cells (**S2B Fig**). We subsequently infected 10-week-old female BALB/c mice with 10^5^ plaque-forming units (PFUs) of each mutant virus and evaluated replication in the lung 2 days postinfection. For WT, MA1, and MA2, no evidence of viable infection was detected in mouse lung tissues (**S2C Fig**); however, MA4 and CMA1-3 had robust replication in mouse lung, suggesting that multiple combinations of RBD changes could provide compatibly with mouse ACE2 sufficient for replication in a standard laboratory mouse strain.

To further evaluate the mouse-adapted strains, we focused on SARS-CoV-2 CMA1, CMA2, and CMA3 mutants over a 4-day time course. In female 10-week-old BALB/c mice infected with 10^5^ PFU, none of 3 mutants induced major disease (**S3A Fig**), although both CMA2 and CMA3 caused more weight loss than CMA1. Examining viral replication in the lung, all 3 mutants produced approximately 10^5^ PFU per lobe at day 2 postinfection (**S3B Fig**). However, no virus was detected at day 4, suggesting rapid clearance by the host. To determine if type I interferon was the major factor blunting infection, IFNAR^−/−^ SJV129 mice were infected with CMA1, CMA2, and CMA3 at 10^5^ PFU. Following infection, all 3 CMA mutant strains caused significant disease, with both CMA2 and CMA3 peaking at approximately 10% weight loss (**S3C Fig**). However, despite increased disease, viral titers were only slightly higher at day 2 than immune competent BALB/c mice and still cleared by day 4 for all 3 strains (**S3D Fig**). Together, the results indicate that SARS-CoV-2 CMA1, CMA2, and CMA3 can replicate in both BALB/c and IFNAR^−/−^ mice, but fail to sustain continued replication in vivo.

### Serial passage of SARS-CoV-2 CMA3

**Fig 1:**
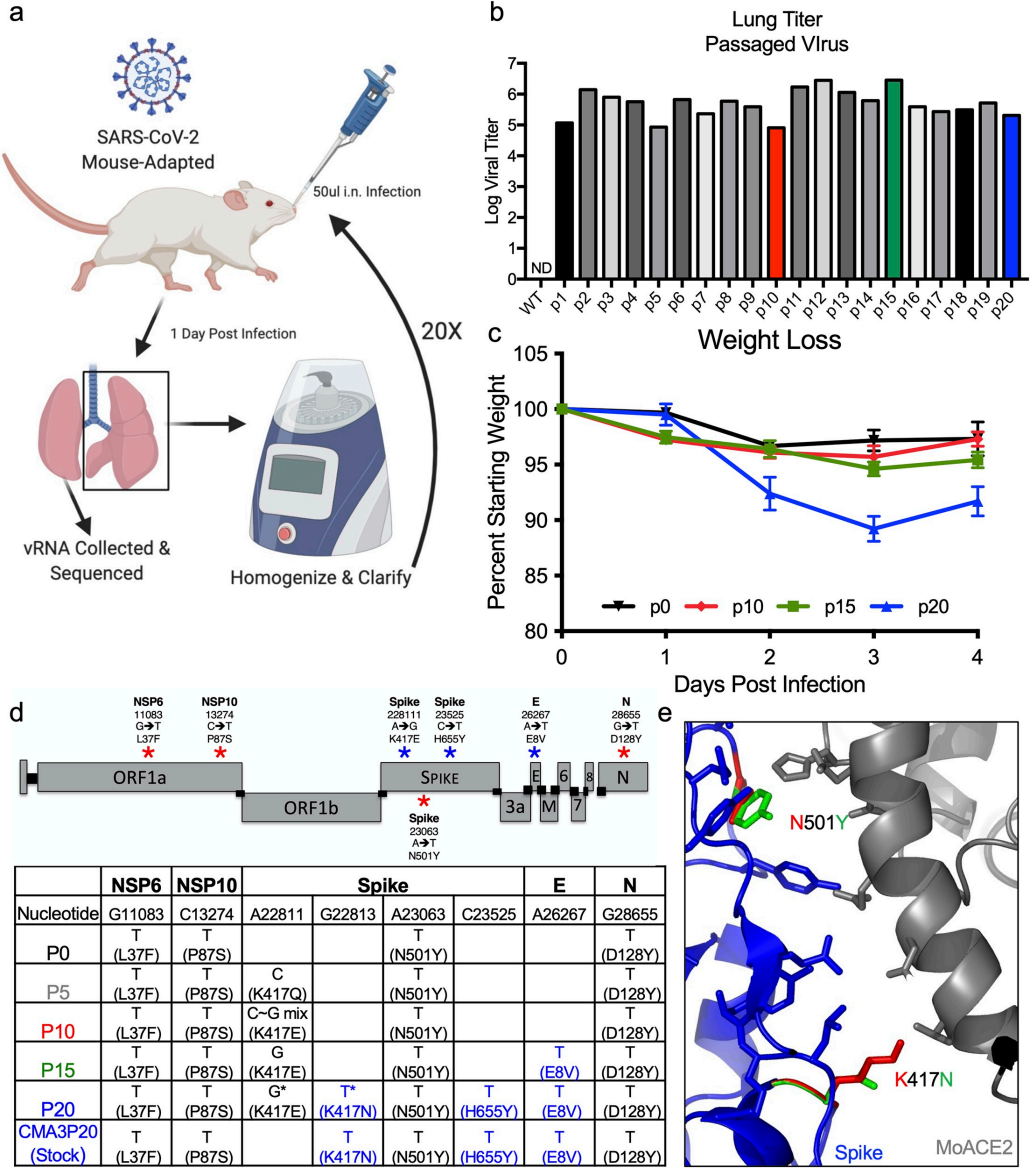
Mouse adaptation of SARS-CoV-2. **(a)** Schematic of adaptation of SARS-CoV-2 CMA3p20. One 10-week-old female BALB/c mice was infected with SARS-CoV-2 CMA3 for 1 day, euthanized, and lung tissues harvested for viral RNA and viral titer determination. Lung tissues were homogenized, clarified, and 50 ul used to inoculate subsequent animals for 20 p. The figure was generated using BioRender software. **(b)** Viral replication of CMA3 p1-p20 from lung homogenates isolated from infected mice 1 day postinfection (*n* = 1). **(c)** Stock virus generated at p0, p10, p15, and p20 was used to infect female BALB/c mice at 10^5^ PFU and evaluated for weight loss over a 4-day time course (*n* = 5). **(d)** Schematic of engineered (red stars) and passage-acquired (blue stars) mutations in CMA3p20 stock virus. Table includes Sanger equivalent accumulation of mutations over p5, p10, p15, p20, and final stock used for subsequent studies. **(e)** Modeling RBD spike mutations N501Y and K417N found in CMA3p20 with mouse ACE2. Model constructed using Pymol 2.4.2. Data presented as mean values +/− SEM in (c). Raw data are available in S1 Data. ACE2, angiotensin converting enzyme 2; p, passage; PFU, plaque-forming unit; RBD, receptor-binding domain; SARS-CoV-2, Severe Acute Respiratory Syndrome Coronavirus 2.

In order to generate a SARS-CoV-2 strain that produced significant disease in an immune competent mouse, we serially passaged SARS-CoV-2 CMA3 in 10-week-old BALB/c mice. A single mouse was infected with 10^5^ PFU of CMA3 (p0); the mouse was subsequently euthanized at 1 day postinfection with half the lung lobes taken for viral RNA and the other lobes homogenized, clarified, and used to inoculate subsequent passages (**Fig 1A**); lung samples were titered by plaque assay to verify continued SARS-CoV-2 replication (**Fig 1B**). After passages (p) 10, p15, and p20, stock viruses were generated on Vero E6 cells, used to infect 10-week-old BALB/c mice, and compared to the disease caused by the original CMA3 p0 strain (**Fig 1C**). Following 10^5^ PFU challenge, mice infected with p10 and p15 were found to have augmented weight loss compared to p0; however, mice infected with p20 showed 10% weight loss by day 3 and signs of disease including ruffled fur and hunched posture. We subsequently deep sequenced the passaged virus from the lung RNA and identified 2 additional spike mutations (K417N and H655Y) and a mutation in the E protein (E8V). Several other mutations were also found as minority variants in the spike and in other parts of the genome **(Fig 1D**). Modeling the RBD interaction (**Fig 1E**), K417N and N501Y likely improve binding to mouse ACE2 and facilitate increased in vivo disease. Similarly, H655Y may play a role in changes to proteolytic processing of the spike protein following receptor binding [15]. Together, mouse adaptation of SARS-CoV-2 CMA3 incorporated 3 additional fixed mutations that drive increased disease in mice.

### Characterization of CMA3p20

Having observed significant disease in mice infected with CMA3p20 relative to the initial strain of CMA3, we next evaluated weight loss, viral replication, and histopathology in BALB/c mice. First, we tested CMA3p20 for a dose-dependent impact on weight loss (**S4A Fig**); both 10^6^ and 10^5^ PFU caused significant, dose-dependent weight loss with minimal disease observed in the 10^4^ challenge. We also compared CMA3p20 infection associated weight loss to a B.1.1.7 SARS-CoV-2 variant (United Kingdom) that contains the N501Y mutation that permits virus replication in mice (**S3A–S3D Fig**). After challenge with the 10^6^ PFU of the B.1.1.7 variant, female BALB/c mice lost approximately 10% of their starting weight by day 2 and recovered (**S4B Fig**). Together, the results indicate more severe disease with the mouse-adapted CMA3p20 than the B.1.1.7 variant.

**Fig 2:**
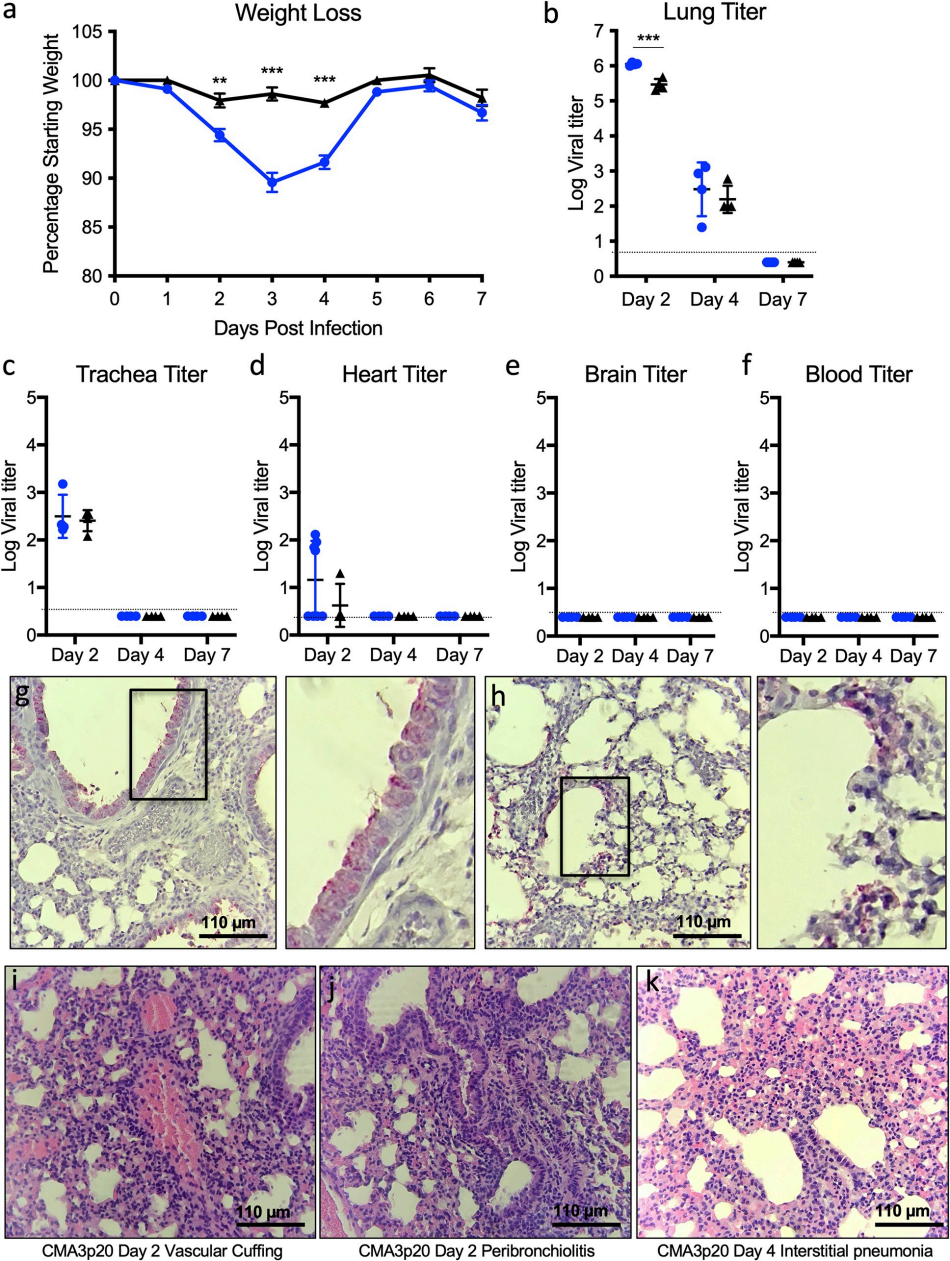
SARS-CoV-2 CMA3p20 induces disease restricted to the lung. **(a–f)** Ten-week-old BALB/c mice were infected with 10^5^ PFU of SARS-CoV-2 CMA3 (black triangle, *n* = 20) or CMA3p20 (blue circles, *n* = 12) and followed for (a) weight loss and viral titer in the (b) lung, (c) trachea, (d) heart, (e) brain, and (f) blood (*n* = 4). **(g–k)** Histology from CMA3p20-infected mice showed viral antigen (N-protein) staining in the (g) airways and (h) parenchyma at day 2 with high magnification in the insets. Significant lung infiltration, inflammation, and damage was observed at (i and j) day 2 and (k) day 4 postinfection. Images representative of most significant damage observed in lung sections observed in CMA3p20. Data presented as mean values +/− SEM in (a). *P* values based on a 2-tailed Student *t* test. Magnification at 10× for (g and i). Raw data are available in S2 Data. PFU, plaque-forming unit; SARS-CoV-2, Severe Acute Respiratory Syndrome Coronavirus 2.

We subsequently used the 10^5^ PFU dose of CMA3p20 to examine infection compared to SARS-CoV-2 CMA3 over a 7-day time course. Following infection, 10-week-old female BALB/c mice CMA3p20-infected mice lost significant weight over the first 4 days, peaking at day 3 with >10% weight loss (**Fig 2A**). By contrast, the original CMA3 caused minimal weight loss over the course of the 7-day infection. We next examined viral replication in the lung at days 2, 4, and 7 postinfection (**Fig 2B**). CMA3p20 infection had a significant 0.5 log increase in viral load over CMA3 in the lung at day 2; this difference was diminished at day 4 (0.25 log increase, not statically significant), and both virus strains were cleared by day 7 in the lung. We also observed day 2 replication in the trachea of mice that was cleared by day 4 in both CMA3 and CMA3p20 infection (**Fig 2C**). Together, the data demonstrate robust weight loss and clear replication in the mouse respiratory tract.

We next evaluated SARS-CoV-2 replication in nonrespiratory tissues. Following infection, we noted replication in the heart tissue of a subset of animals at day 2 (**Fig 2D**). However, infection was transient and not uniform in all animals, and no virus was detected in the heart at later time points. We subsequently evaluated viral load in the brain and blood and found no evidence for CMA3 or CMA3p20 infection by plaque assay (**Fig 2E and 2F**). To further verify viral replication, we also examined viral RNA expression in the lung, heart, brain, spleen, and liver (**S4C Fig**). While robust viral RNA was observed in the lung, the other tissues had minimal evidence for CMA3p20 replication. Together, the data indicate that the SARS-CoV-2 CMA3p20 strain is primarily restricted to and disease driven by virus replication in the respiratory tract.

### CMA3p20 induces significant immune infiltration and lung damage

Further examining lung tissue, histopathology analysis of CMA3p20 infection indicated robust virus replication, immune infiltration, and tissue damage. Using antigen staining against the N protein, we saw evidence for viral replication primarily in the bronchioles with additional staining in the lung parenchyma at day 2 postinfection (**Fig 2G and 2H**). We also observed lung infiltration and inflammation following CMA3p20 challenge characterized by peribronchioloitis, perivascular cuffing, and perivasculitis by day 2 postinfection (**Fig 2I and 2J, S5A and S5B Fig**). Similarly, at day 4, we noted collapsed airways and interstitial pneumonia (**Fig 2K**). Some portions of the day 4 lungs infected with CMA3p20 also had virus induced damage including enlarged and multinucleated alveolar type II cells (**S5C Fig**) and loss of cellular polarity (**S5D Fig**). Together, the histopathology results demonstrated significant damage, inflammation, and disease in the lung following infection with SARS-CoV-2 CMA3p20.

### CMA3p20 retains replication capacity in primary human respiratory cells

**Fig 3:**
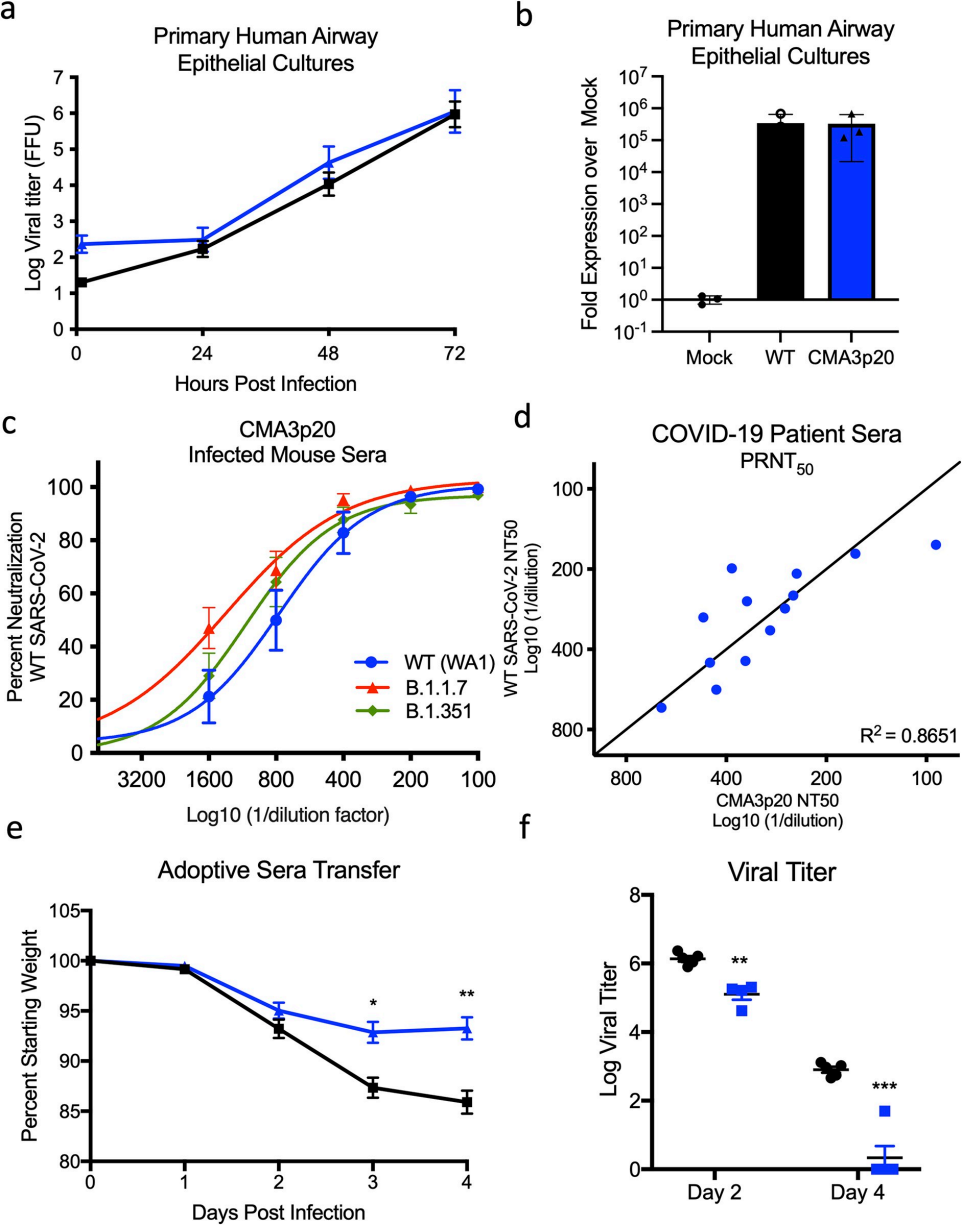
CMA3p20 strain maintains human replication capacity and antigenicity. **(a and b)** Primary human airway cultures were infected with SARS-CoV-2 WT (black square) or CMA3p20 (blue circles) at an MOI of 0.01 and evaluated for (a) viral titer and (b) viral RNA (*n* = 3). **(c)** Sera collected from female BALB/c mice 28 days postinfection with 10^6^ PFU of SARS-CoV-2 CMA3p20 were evaluated for capacity to neutralize WT SARS-CoV-2 (WA1), B.1.1.7, and B.1.351 via PRNT_50_ assay (*n* = 12). Log10 value of dilution used to plot points on the x-axis. **(d)** PRNT_50_ values from COVID-19 patient sera plotted against WT virus (y-axis) versus CMA3p20 virus (x-axis). **(e and f)** Ten-week-old female BALB/c mice were treated intraperitoneally with 100 ul of human COVID-19 sera (*n* = 10) or control (PBS, *n* = 10) 1 day prior to infection. Mice were subsequently challenged with 10^5^ PFU of SARS-CoV-2 CMA3p20 and evaluated for (e) weight loss and (f) viral titer in the lung (*n* = 5). Data presented as mean values +/− SD in (a–c) and +/− SEM in (e). *P* values based on a 2-tailed Student *t* test. Raw data are available in S3 Data. COVID-19, Coronavirus Disease 2019; MOI, multiplicity of infection; PFU, plaque-forming unit; PRNT, plaque reduction neuralization titer; SARS-CoV-2, Severe Acute Respiratory Syndrome Coronavirus 2; WT, wild-type.

Altering SARS-CoV-2 to be permissive in mice can impact its replication capacity in human cells [16]. Therefore, we examined the ability of CMA3p20 to replicate in primary human airway epithelial (HAE) cultures compared with the WT SARS-CoV-2 WA1 strain. Grown on an air–liquid interface, primary HAEs represent a useful in vitro model of the human airway [17]. Following infection, CMA3p20 had equal replication to WT SARS-CoV-2 over a 72-hour time course in primary HAE cultures (**Fig 3A**). Similarly, viral RNA levels at 72 hours postinfection were equivalent between CMA3p20 and SARS-CoV-2 WA1 strain (**Fig 3B**). Together, the results indicate that mouse adaption resulted in no significant replication attenuation of CMA3p20 in primary human airway cells.

### CMA3p20 retains antigenicity similar to WT SARS-CoV-2

In addition to differences in replication in human cells, spike changes in SARS-CoV-2 could alter the overall antigenicity of CMA3p20 as compared to SARS-CoV-2 derived from humans; this result would make it more difficult to interpret vaccine and protection studies derived from mice. Therefore, to evaluate antigenicity, we infected 10-week-old female BALB/c mice with 10^6^ PFU of CMA3p20 and then euthanized and harvested sera 28 days postinfection. We subsequently used the mouse sera to measure plaque reduction neuralization titer (PRNT_50_) against the WT SARS-CoV-2 WA1 strain as well as 2 variants of concern (B.1.1.7 and B.1.351) (**Fig 3C**). Mouse sera from mice (*n* = 12) infected with CMA3p20 neutralized WT SARS-CoV-2 (WA1) and the other SARS-CoV-2 variants of concern with a PRNT_50_ value ranging from 776 for WT, 1312 for B.1.1.7, and 1091 for B.1.351. To further evaluate CMA3p20 antigenicity, we examined PRNT_50_ assays using sera from acutely infected, hospitalized COVID-19 patients (**Fig 3D**). Performing neutralization assays in parallel, we found that CMA3p20 had PRNT_50_ values similar to WT SARS-CoV-2 with each COVID-19 patient serum tested. With a R^2^ value of 0.8651 over the 13 samples, the results indicated that CMA3p20 retains similar antigenicity to the WT SARS-CoV-2 and has potential use for vaccine and protection studies.

To further demonstrate the use of CMA3p20 to understand in vivo protection, we performed a passive transfer experiment with a COVID-19 patient serum. One day prior to infection, 10-week-old female BALB/c mice were pretreated intraperitoneally with either control (PBS) or 100 ul of serum from a COVID-19 patient with a neutralization titer of 1,230. Mice were subsequently challenged with 10^5^ PFU of CMA3p20 and monitored for weight loss and viral titer. Mice treated with acutely infected COVID-19 patient serum had significantly reduced weight loss at day 3 and 4 postinfection as compared to control mice (**Fig 3E**). Similarly, viral titers in the lung were reduced at both day 2 and day 4 in mice receiving COVID-19 patient serum as compared to control. Consistent with the PRNT_50_ titer (**Fig 3D**), the results from the passive transfer experiment demonstrate that antibody-based immunity generated following human infection can effectively neutralize SARS-CoV-2 CMA3p20. Together, the results confirm similar antigenicity of CMA3p20 and WT SARS-CoV-2.

### Prior SARS-CoV-2 infection protects from lethal SARS-CoV challenge

**Fig 4:**
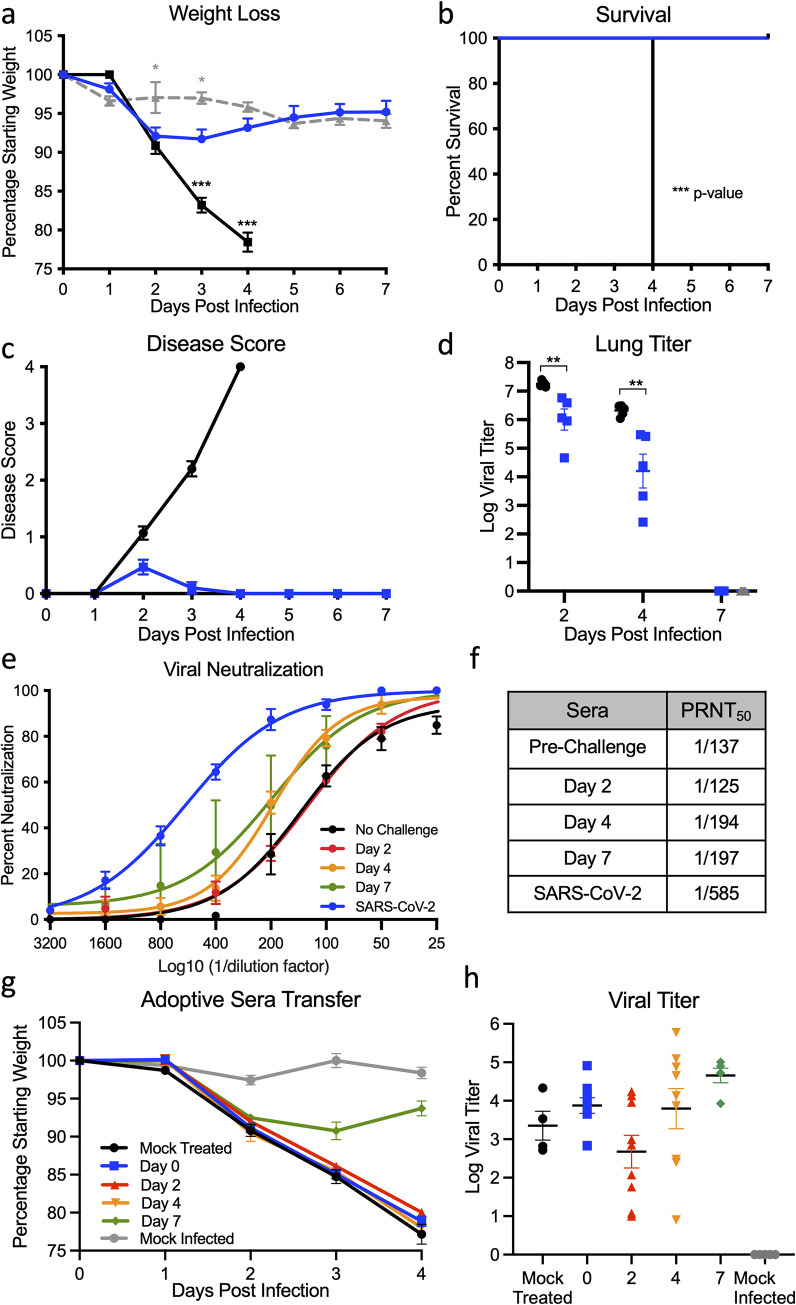
Prior infection with SARS-CoV-2 protects from lethal SARS-CoV challenge. **(a–c)** Ten-week-old female BALB/c mice were previously infected with 10^6^ PFU of SARS-CoV-2 CMA3p20 (blue line, *n* = 15) or mock infected (black line, *n* = 15), monitored for weight loss, and allowed to recover. Moreover, 28 days postinfection, both groups were challenged with a lethal dose (10^4^ PFU) of mouse-adapted SARS-CoV or SARS-CoV-2 recovered animals mock infected with PBS (gray dotted line) and evaluated for (a) weight loss, (b) lethality, and (c) disease score. **(d)** Mice were subsequently euthanized on days 2, 4, and 7 and lung tissue examined for viral replication (*n* = 5). **(e and f)** Sera from CMA3p20 infected and SARS-CoV challenged were evaluated for (e) virus neutralization (PRNT_50_) against SARS-CoV-2 (blue) or SARS-CoV over time (no rechallenge: black, day 2: red, day 4: orange, and day 7: green, *n* = 5) and (f) PRNT_50_ titer reported as reciprocal values. Log10 value of dilution used to plot points on the x-axis in e. **(g and h)** Sera from CMA3p20 infected and SARS-CoV challenged was adoptively transferred to naive 10-week-old BALB/c mice (mock transfer: black, *n* = 5; day 0: blue, *n* = 10; day 2: red, *n* = 10; day 4: orange, *n* = 10; and day 7: green, *n* = 5), which were subsequently challenged with mouse-adapted SARS-CoV. Animals were monitored for (g) weight loss and (h) viral replication in the lung following infection. Data presented as mean values +/− SEM in (a) and +/− SD in (e). *P* values based on a 2-tailed Student *t* test. Raw data are available in S4 Data. PRNT, plaque reduction neuralization titer; SARS-CoV, Severe Acute Respiratory Syndrome Coronavirus; SARS-CoV-2, Severe Acute Respiratory Syndrome Coronavirus 2.

Having established a SARS-CoV-2 mouse model with significant disease, we next evaluated the capacity of CMA3p20 to protect against heterologous SARS-CoV challenge. Ten-week-old female BALB/c mice were infected with 10^6^ PFU of CMA3p20 or control (PBS), monitored for weight loss, and allowed to recover. CMA3p20-infected and control mice were subsequently challenged with a lethal dose of mouse-adapted SARS-CoV (10^4^ PFU) [18]. Control mice infected with SARS-CoV MA15 had rapid weight loss and lethality with all mice reaching euthanasia criteria by day 4 postinfection (**Fig 4A and 4B**). By contrast, mice previously infected with SARS-CoV-2 CMA3p20 had less weight loss compared to controls, only losing approximately 10% of their starting weight by day 2. The CMA3p20-infected mice recovered their starting weight at late times, demonstrating protection from lethal SARS-CoV infection. Examining disease score, mice previously infect with SARS-CoV-2 CMA3p20 showed some disease at day 2, but were generally devoid of ruffled fur, diminished movement, or hunching (**Fig 4C**). By contrast, the mock infected animals challenged with SARS-CoV showed significant disease that escalated over the course of infection and required euthanasia by day 4 for all animals remaining in the study (*n* = 10). For both CMA3p20-infected and CMA3p20-uninfected animals, robust SARS-CoV replication was observed in the lung (**Fig 4D**). However, mice infected with CMA3p20 has a significant reduction in viral loads as compared to control animals. Yet, the viral replication indicates sterilizing immunity was not achieved.

We subsequently evaluated the neutralization capacity of SARS-CoV-2 CMA3p20 sera against SARS-CoV following challenge (**Fig 4E**). CMA3p20 mouse sera from prechallenge, day 2, day 4, and 7 post–SARS-CoV challenge were able to neutralize SARS-CoV with a low range of PRNT_50_ titers (**Fig 4F**). Both the prechallenge and day 2 postchallenge sera had neutralization titers approximately 150, while day 4 and 7 postchallenge sera were only augmented to approximately 200. While significantly less neutralization than what is observed against WT SARS-CoV-2 (approximately 600), the results suggest that SARS-CoV-2 CMA3p20 infection induces protection sufficient to protect from lethal challenge with SARS-CoV.

To further examine the immune response, we next evaluated the role of antibodies in conferring protection via a serum transfer experiment. Briefly, sera from mice previously infected with SARS-CoV-2 CMA3p20 and challenged with SARS-CoV (**Fig 4A–4D**) was transferred via intraperitoneal injection to naive mice; these mice were subsequently infected with SARS-CoV MA15 and monitored for weight loss. Consistent with the above infection, mock-treated animals had rapid weight loss following SARS-CoV infection (**Fig 4G**). However, contrasting the rechallenge study, adoptive sera transfer from SARS-CoV-2–infected animals offered no protection from weight loss and disease. Similarly, sera from SARS-CoV-2 primed animals 2 and 4 days post–SARS-CoV challenge also failed to alter weight loss despite the presence of low neutralizing antibody titer. Only sera from SARS-CoV-2–infected animals 7 days post–SARS-CoV challenge offered any protection; yet, there was still significant weight loss (approximately 10%), suggesting incomplete protection by antibody alone. Importantly, despite some protection with day 7 sera, viral titer in the lung was detected in all the infected treatment groups (**Fig 4H**). A limitation of these studies is that the amount of sera capable of transfer is limited to a maximum volume that may not reach the necessary levels to provide protection. Alternatively, the results argue that protection from heterologous SARS-CoV challenge is not conveyed by circulating antibody alone and other elements of adaptive immunity contribute to protection.

## Discussion

In this manuscript, we used a reverse genetic system [12] and in vivo adaptation to generate a mouse-adapted strain of SARS-CoV-2 (CMA3p20). CMA3p20 induces a dose-dependent disease in young, female BALB/c mice with viral replication limited primarily to the respiratory tract. In addition, CMA3p20 infection causes substantial damage to the mouse lung with significant inflammation, immune infiltration, and pneumonia. Importantly, mutations in CMA3p20 do not alter its ability to infect primary human cell airway cells, and the mouse-adapted strain maintains similar antigenicity to WT and variant SARS-CoV-2. Using this model, we demonstrated that infection with SARS-CoV-2 CMA3p20 provided protection from lethal challenge with the mouse-adapted SARS-CoV, and this protection is not conveyed by circulating antibody alone. Together, results indicate the use of CMA3p20 as a model to study SARS-CoV-2 pathogenesis and immunity in standard inbred mice.

With the threat posed by SARS-CoV-2 and its emerging variants, questions have been raised on the efficacy and duration of immunity following natural infection [19,20]. Based on the protection provided by prior SARS-CoV-2 challenge against the heterologous SARS-CoV, our results suggest that immunity is more complex than neutralizing serum titer alone. While sera from mice challenged with SARS-CoV-2 CMA3p20 neutralizes SARS-CoV at approximately 150, this level of antibody does not provide sterilizing immunity in the lung. Even after SARS-CoV infection, the serum neutralization is only approximately 200 at day 7 postinfection. Consistent with this finding, adoptive serum transfer failed to provide protection until late times post–SARS-CoV rechallenge. While the transferred antibody titers were similar between day 4 and day 7 post-SARS-CoV challenge, the distinction in outcomes may be due differences to antibody class (immunoglobulin M [IgM] versus immunoglobulin G [IgG]) or in antibody production in the tissue versus the blood. In terms of weight loss and disease, the SARS-CoV-2–infected mice showed a significant reduction in severity and complete protection from SARS-CoV–induced lethality. Overall, the results suggest that components of the host adaptive immune response in conjunction with antibodies contribute to protection from heterologous challenge.

From these initial findings, the exact mechanism of protection is not yet clear. With previous studies with SARS-CoV, antibody-based immunity has led to complete protection from viral replication in the lung [21,22]. Similar findings have been observed in animal models of SARS-CoV-2, leading to serum neutralization as a primary correlate associated with protection [23]. However, studies with SARS-CoV have also implicated a role for cellular-based immunity [24]. Notably, initial findings have found that non-neutralizing antibody responses against common cold CoV had some level of protection [25]. Importantly, the vast majority of T-cell studies have found epitopes directed against the more conserved nucleocapsid protein [25]. Absent in most vaccine platforms, it is unclear if the immunity stimulated by natural SARS-CoV-2 infection will mimic vaccine induced immunity and offer protection against heterologous SARS-CoV challenge.

In regard to the mouse-adapted strain, CMA3p20, several of the key mutations in the spike protein have been observed in novel SARS-CoV-2 variants of concern [26]. Starting with the spike mutations, N501Y is represented in several COVID-19 variants of concern, found in 29% of the GISAID database of reported sequences, and has been shown to improve binding to human ACE2 [27]. In these studies, N501Y alone (CMA1) permits SARS-CoV-2 to replicate in BALB/c mice. Similarly, spike mutation K417N, also found in several variants of concern, likely augments receptor binding and drives in vivo disease [28,29]. In contrast to the other spike mutations, H655Y is outside the RBD and has low penetrance in GISAID sequences (<0.5%) [28]. However, recent studies have indicated that the H655Y mutations impacts to proteolytic processing of the spike and may alter infection [15]. Outside the spike changes, the other mutations found in CMA3p20 are not clear in their impact. Mutations in NSP8, NSP10 (<0.5%), and E protein are rarely found in GISAID reported sequences, are not in conserved domains, and may be hitchhiking mutations [30]. Notably, NSP6 (L37F) has been in observed in 4% of human SARS-CoV-2 isolates, suggesting possible selection [30]. Similarly, while N change at 128 is not common [28], differences in disease between CMA2 and CMA1 suggest a role in pathogenesis. Finally, despite significant variation across the Sarbecovirus family, no mutations were observed in the mouse-adapted strain in the accessory proteins.

While the mouse-adapted mutations in CMA3p20 render a pathogenic virus in mice, it does not ablate the replication capacity in human cells or antigenicity relative to the WT SARS-CoV-2 strains. Previously described mouse-adapted strains have been shown to cause significant disease, but lose replication capacity in primary human airway cultures [16]. In these studies, CMA3p20 has similar replication levels as SARS-CoV-2 WA1 in primary HAE cultures. In addition, CMA3p20-infected mice generate antibody responses capable of neutralizing WT SARS-CoV-2 WA1 as well as the B.1.1.7 and B.1.351 variants. In addition, the mouse-adapted strain is similarly neutralized by COVID-19 sera. Importantly, passive transfer of human convalescent sera reduced disease in mice challenged with SARS-CoV-2, although protection was incomplete potentially be due possibly to inefficient transfer or incompatible effector functions between mice and humans. Together, the results indicate that CMA3p20 induces a robust immune response similar to that seen in humans and useful for understanding immunity in standard animal models.

## Methods

### Viruses and cells

The recombinant WT and mouse-adapted strains of SARS-CoV-2 are based on the sequence of USA-WA1/2020 isolate provided by the World Reference Center for Emerging Viruses and Arboviruses (WRCEVA) and was originally obtained from the United States of America Centers for Disease Control and Prevention as described [31]. SARS-CoV B.1.1.7 isolate used in this study was provided to the WRCEVA by Dr. Natalie Thornburg and colleagues at the Centers for Disease Control and Prevention. WT and mutant SARS-CoV-2 as well as recombinant mouse-adapted recombinant SARS-CoV [18] were titrated and propagated on Vero E6 cells, grown in DMEM with 5% fetal bovine serum and 1% antibiotic/antimycotic (Gibco, Amarillo, TX). Standard plaque assays were used for SARS-CoV and SARS-CoV-2 [32,33]. All experiments involving infectious virus were conducted at the University of Texas Medical Branch (UTMB; Galveston, Texas, USA) or Emory University (Atlanta, Georgia, USA) in approved Biosafety Level (BSL) 3 laboratories with routine medical monitoring of staff.

### Phylogenetic tree, sequence identity heat map, and structural modeling

Spike receptor binding tables were constructed from a set of representative group 2B coronaviruses by using alignment data paired with neighbor-joining phylogenetic trees built in Geneious (v.9.1.5) using the spike amino acid sequences derived the following accession numbers: QHU79204 (SARS-CoV-2 WA1), AGZ48806 (RsSHC014), ALK02457 (WIV16), and AYV99817.1(SARS-CoV Urbani). Mouse-adapted SARS-CoV-2 structural homology models were generated using SWISS-Model [34, 35] with the 6LZG crystal structure (RCSB Protein Data Bank) as the template structure for the spike protein and the 2AJF crystal structure (RCSB Protein Data Bank) as the template for ACE2. Homology models were visualized and manipulated in PyMOL (version 2.4.2).

### Construction of mouse-adapted mutant SARS-CoV-2

Both WT and mutant viruses were derived from the SARS-CoV-2 USA-WA1/2020 infectious clone as previously described [12]. For mouse-adapted virus construction, the individual mutations were synthesized and introduced into the appropriate plasmids (F1 to F7) via PCR-based mutagenesis with synthesized specific primers containing corresponding mutations. The resulted plasmid was validated by further restriction enzyme digestion and Sanger sequencing. Thereafter, plasmids containing WT and mutant SARS-CoV-2 genome fragments were amplified and digested by restriction enzyme. The SARS-CoV-2 genome fragments were purified and ligated in vitro to assemble the full-length cDNA according to the procedures described previously [12,36]. In vitro transcription reactions then were performed to synthesize full-length genomic RNA. To recover the viruses, the RNA transcripts were electroporated into Vero E6 cells. The medium from electroporated cells as harvested at 40 hours posttransfection and served as seed stocks for amplifying one passage on Vero E6 cells (P1 stock). Viral mutants were confirmed by sequence analysis prior to use. Synthetic construction of SARS-CoV-2 mouse-adapted strains were approved by the UTMB Institutional Biosafety Committee.

### In vitro infection

Viral infections in primary human airway cells were performed as previously described [37]. Briefly, the apical side of the HAE cultures was washed 3 times with PBS. Cultures were infected with SARS-CoV-2 WT (WA1) or CMA3p20 at MOI 0.01 and allowed to adsorb for 1 hour at 37°C. After adsorption, the apical side was washed 3 times with PBS, and the basolateral media was replaced. Viral washes were collected adding PBS to the apical side and incubated for 30 minutes at 37°C. Viral titer was evaluated by focus forming assay as previously described [37]. RNA from HAE were collected at 48 hours postinfection. RNA was harvested from mock-infected and infected HAE cultures by treating with RNA lysis buffer for >5 minutes and gently pipetting to recover cells. Total RNA was extracted using the Zymo Quick-RNA miniprep kit (VWR (Radnor, PA); R1055) according to the manufacturer’s protocol. Purified RNA was reverse transcribed into cDNA using the high-capacity cDNA reverse transcription kit (Thermo Fisher Scientific (Sugarland, TX), 43-688-13). RNA levels in HAE cells were quantified using the IDT Prime Time gene expression master mix and TaqMan gene expression Primer/Probe sets (IDT, Coralville, IA) and run on a QuantStudio5 qPCR system using SARS-CoV-2 RDRP-specific primers (forward [F], GTGARATGGTCATGTGTGGCGG; reverse [R], CARATGTTAAASACACTATTAGCATA) and probe (56-6-carboxyfluorescein [FAM]/CAGGTGGAA/ZEN/CCTCATCAGGAGATGC/3IABkFQ) were used. A total of 3 or more biological replicates were harvested at each described time, and results are representative of multiple experiments. No blinding was used in any sample collections, nor were samples randomized. Microsoft Excel for Mac 2011 was used to analyze data.

### Deep sequencing analysis

RNA libraries of SARS-CoV-2 mutants were prepared with 300 ng of RNA using the Tiled-ClickSeq protocol as previously described [38,39] using tiled primers cognate to the SARS-CoV-2 genome (accession number NC_045512.2) and the TruSeq i7 LT adapter series and i5 hexamer adaptors containing a 12N unique molecular identifier (UMI). Libraries were sequenced on the Illumina MiSeq platform with MiSeq Reagent Kit v2 using paired-end reads (R1:250 cycles, R2:50 cycles). Raw data were demultiplexed using TruSeq indexes using the MiSeq Reporter Software. Demultiplexed read data were quality filtered, adaptor trimmed, and primer trimmed as previously described [39]. Reads were mapped to the WA-1 reference (NC_045512.2) using *ViReMa* [40]. Reads were deduplicated with *umi_tools* [41] using 12N UMIs embedded in the i5 click-adaptor. A consensus reference sequence was generated using Pilon [42], ensuring that read coverage was greater than 25× across 99.5% of the reference genome. Pileup files were generated using Samtools v1.9 [43], and minority variants were extracted by nucleotide voting (PHRED> = 30) using a custom Python 3 script previously described [39].

### Plaque reduction neutralization test

Neutralization assays were preformed using conventional plaque reduction neutralization assay (PRNT_50_) as previously described [44]. Briefly, 100 PFU of SARS-CoV-2, mouse-adapted SARS-CoV-2, or SARS-CoV MA15 was incubated with serially diluted serum from mice or COVID-19 patients (total volume of 200 μl) at 37°C for 1 hour. The virus–serum mixture was added to the preseeded Vero E6 cells. After 1 hour 37°C incubation, 2 ml of 2% high gel temperature agar (SeaKem) in DMEM containing 5% FBS and 1% P/S was overlaid onto infected cells. After 2 days of incubation, 2 ml neutral red (1 g/l in PBS; Sigma, St. Louis, MO) was added to the agar-covered cells. After another 5-hour incubation, neutral red was removed. Plaques were counted for NT50 calculation, which were determined by using arithmetic mean and the Sigmoidal, 4PL function in PRISM 9 software. The PRNT_50_ assay was performed at the BSL-3 facility at UTMB.

### Ethic statement

This study was carried out in accordance with the recommendations for care and use of animals by the Office of Laboratory Animal Welfare, National Institutes of Health. The Institutional Animal Care and Use Committee (IACUC) of the UTMB approved the animal studies under protocols 1711065 and 1707046. For samples Emory University, collection and processing were performed under approval from the University Institutional Review Board (IRB #00001080 and #00022371). Adults ≥18 years were enrolled who met eligibility criteria for SARS-CoV-2 infection (PCR or rapid antigen test confirmed by a commercially available assay) and provided informed consent.

### Human serum samples

For Emory University, acute peripheral blood samples were collected from hospitalized patients at the time of enrollment. Infected patients were randomly selected from a convenience sample, and no data were collected on the number of patients that were prescreened or declined participation. All patients enrolled in July 2020 and had a mean age of 57 (range: 26 to 85; 50% male). Samples were collected in the first 9 days (range: 2 to 9) of their hospital stay (range: 3 to 33 days) and mostly 1 to 2 weeks after symptom onset (range 5 to 19 days), the majority of the patients had comorbid conditions (*n* = 16), with 19 out of 20 having severe disease and 1 patient had moderate disease. All of these patients had radiological evidence of pneumonia; 19 out of the 20 patients required supplemental oxygen, and 4 out of 20 patients were admitted to the intensive care unit (ICU). A total of 3 enrolled patients died of COVID-19.

### Mice and in vivo infection

Ten-week-old BALB/c mice were purchased from Charles River Laboratories (Wilmington, MA) and were maintained in Sealsafe HEPA-filtered air in/out units. Prior to infection, animals were anesthetized with isoflurane and infected intranasally (IN) with 10^4^ to 10^6^ PFUs diluted 50 μl of PBS. Infected animals were monitored for weight loss, morbidity, and clinical signs of disease, and lung titers were determined as described previously [45]. Briefly, the right, lower lobe of the mouse lung was homogenized in 1 mL of PBS, clarified by centrifugation, and titered by serial 10-fold dilution on VERO E6 cells; titers reflective of PFU per lobe of lung. Infected animals were weighed daily, and lung tissue was collected 2, 4, and 7 days postinfection for downstream analysis by plaque assay.

### Real-time PCR for in vivo viral RNA

RNA from tissues were collected using RNA later [46]. Samples were subsequently homogenized with Trizol reagent (Invitrogen, Carlsbad, CA). RNA was then extracted from Triazol using the Direct-zol RNA Miniprep Plus kit (Zymo Research (Irvine, CA), #R2072) per the manufacturer’s instruction. Extracted RNA was then converted to cDNA with the iScript cDNA Synthesis kit (Bio-Rad (Hercules, CA), #1708891). Quantitative real time PCR (qRT-PCR) was performed with the Luna Universal qPCR Master Mix (New England Biolabs (Ipswich, MA), #M3003) on a CFX Connect instrument (Bio-Rad #1855200). Primer 1 (Forward—AAT GTT TTT CAA ACA CGT GCA G) and Primer 2 (Reverse—TAC ACT ACG TGC CCG CCG AGG) were used to detect SARS-CoV-2 genomes as previously described [47]. A primer annealing temperature of 63°C was used for all assays.

### Histological analysis

The left lung was removed and submerged in 10% buffered formalin (Fisher, Waltham, MA) without inflation for 1 week. Lungs from mice sacrificed on day 2 and day 4 were fixed in formalin and paraffin embedded. Moreover, 5-μm serial sections were taken and used for histopathological staining with hematoxylin and eosin and immunohistochemistry (IHC) assay. IHC was conducted using rabbit polyclonal antibodies raised against the SARS-CoV nucleocapsid protein (clone NB100-56576 [1:100], Novus Biologicals, Littleton, Colorado, USA) and biotinylated anti-rabbit IgG, streptavidin AP (Vector Labs, Burlingame, California, USA) with Permanent Red chromogen (Dako/Agilent, Santa Clara, California, USA). Normal rabbit serum was used as primary antibody for the negative control. Briefly, the sections were deparaffinized by immersion in 3 xylene baths for 5 minutes each. The slides were rehydrated by immersion in a series of alcohol baths ranging from 100 to 95% for 5 minutes each. The slides were pretreated with a citrate-based buffer, pH 6.0 at 98° C for heat-induced epitope retrieval. Endogenous avidin and biotin sites were blocked using avidin/biotin blocking kit (Abcam, Cambridge, Massachusetts, USA). The slides were incubated with anti-SARS-CoV antibody for 1 hour at room temperature, washed in 1X TBS, 0.05% Tween 20 (wash buffer), and incubated with goat biotinylated anti-rabbit IgG (H + L) antibody for 30 minutes at room temperature followed by rinse in wash buffer. Slides were then incubated with streptavidin-AP conjugate for 30 minutes at room temperature and washed, followed by incubation with Permanent Red for 5 minutes. Counterstaining with Mayer’s hematoxylin solution was performed and the slides mounted with coverslip using Permount mounting medium.

The raw data that support the findings of this study are available from the corresponding author upon request [48].

### Biological materials

Recombinant WT and mutant SARS-CoV-2 described in this manuscript will be made available through the WRCEVA at UTMB through material transfer agreement and verification of proper containment facilities. The SARS-CoV-2 B.1.1.7 strain was provided by Dr. Natalie Thornburg and colleagues at the Centers for Disease Control and Prevention.

## Supporting information

S1 Fig Modeling changes to mouse-adapt SARS-CoV-2. **(a)** Key amino acid residues found in the RBD of mouse-adapted strains of SARS-CoV were aligned to SARS-CoV-2 and used to design mouse-adapted mutations [13]. Key interaction sites between SARS-CoV spike and ACE2 molecules highlight in red [49]. **(b and c)** Modeling of key RBD residue interactions with mouse ACE2 (PDB:2AJF) comparing (b) WT SARS-Cov-2 residues versus (c) mutations (green) predicted to improve binding. ACE2, angiotensin converting enzyme 2; RBD, receptor-binding domain; SARS-CoV, Severe Acute Respiratory Syndrome Coronavirus; SARS-CoV-2, Severe Acute Respiratory Syndrome Coronavirus 2; WT, wild-type.
(TIFF)

S2 Fig Construction of mouse-adapted SARS-CoV-2 mutants. **(a)** SARS-CoV-2 genome schematic indicating location of amino acid mutations for MA1, MA2, MA4, CMA1, CMA2, and CMA3. **(b)** Viral replication of stock viruses of MA1, MA2, MA4, and CMA1-3 grown on VeroE6 cells. **(c)** Viral replication of MA1, MA2, MA4, and CMA1-3 from lung homogenates isolated from infected mice 2 days postinfection (*n* = 1). Raw data are available in S5 Data. SARS-CoV-2, Severe Acute Respiratory Syndrome Coronavirus 2.
(TIFF)

S3 Fig SARS-CoV-2 mutants CMA1, CMA2, and CMA3 replicate in laboratory mice. **(a and b)** Ten-week-old female BALB/c mice infected with 10^5^ PFU of CMA1 (red), CMA2 (green), or CMA3 (black) were examined for (a) weight loss (*n* = 6) and (b) viral lung titer following infection at days 2 and 4 (*n* = 3). **(c and d)** Ten- to 12-week-old female IFNAR^−/−^ SVJ129 mice infected 10^5^ PFU of CMA1 (red), CMA2 (green), or CMA3 (black) were examined for (c) weight loss (*n* = 6) and (d) viral lung titer following infection at days 2 and 4 (*n* = 4). Data presented as mean values +/− SEM in (a and c). Raw data are available in S6 Data. ND, nondetected; PFU, plaque-forming unit; SARS-CoV-2, Severe Acute Respiratory Syndrome Coronavirus 2.
(TIFF)

S4 Fig In vivo characterization of SARS-CoV-2 CMA3p20. **(a)** Examination of 10-week-old female BALB/c mice infected with SARS-CoV-2 CMA3p20 at 10^4^, 10^5^, and10^6^ PFU (*n* = 5). **(b)** Comparison of weight loss in 10-week old female BALB/c mice infected with 10^6^ PFU of SARS-CoV-2 CMA3p20 (blue) or SARS-CoV-2 variant B.1.1.7 (orange). **(c)** RT-PCR of viral RNA load found in lung, heart, brain, spleen, liver, and kidney following 10^5^ PFU infection of SARS-CoV-2 CMA3p20 2- and 4-days postinfection. Dotted line signifies viral RNA value derived from mock infected samples. Data presented as mean values +/− SEM in (a–c). Raw data are available in S7 Data. PFU, plaque-forming unit; RT-PCR, real time PCR; SARS-CoV-2, Severe Acute Respiratory Syndrome Coronavirus 2.
(TIFF)

S5 Fig SARS-CoV-2 CMA3p20 induces significant lung damage following infection. **(a–c)** CMA3p20-infected animals 2 days postinfection showing (a) perivasculitis and (b) peribronchiolitis. **(c and d)** CMA3p20 induced lung inflammation and damage 4 days postinfection including (c) cytopathic effect of the virus and (d) loss of cellular polarity as indicated by the black arrows. Magnification at 10× for (a–d). SARS-CoV-2, Severe Acute Respiratory Syndrome Coronavirus 2.
(TIFF)

S1 Data Raw data for Fig 1. (XLSX)

S2 Data Raw data for Fig 2. (XLSX)

S3 Data Raw data for Fig 3. (XLSX)

S4 Data Raw data for Fig 4. (XLSX)

S5 Data Raw data for S2 Fig. (XLSX)

S6 Data Raw data for S3 Fig. (XLSX)

S7 Data Raw data for S4 Fig. (XLSX)

## Abbreviations

ACE2: angiotensin converting enzyme 2
BSL: Biosafety Level
CoV: Coronavirus
COVID-19: Coronavirus Disease 2019
HAE: human airway epithelial
IACUC: Institutional Animal Care and Use Committee
ICU: intensive care unit
IgG: immunoglobulin G
IgM: immunoglobulin M
IHC: immunohistochemistry
IN: intranasally
IRB: Institutional Review Board
MERS-CoV: Middle East Respiratory Syndrome Coronavirus
p: passage
PFU: plaque-forming unit
PRNT: plaque reduction neuralization titer
qRT-PCR: quantitative real time PCR
RBD: receptor-binding domain
SARS-CoV: Severe Acute Respiratory Syndrome Coronavirus
SARS-CoV-2: Severe Acute Respiratory Syndrome Coronavirus 2
UMI: unique molecular identifier
UTMB: University of Texas Medical Branch
WRCEVA: World Reference Center for Emerging Viruses and Arboviruses
WT: wild-type
