# Supplementary figures and images for: Mouse-adapted SARS-CoV-2 protects animals from lethal SARS-CoV challenge
Source: PLoS Biol. 2021 Nov 4;19(11):e3001284. doi: 10.1371/journal.pbio.3001284 (PMC8594810; doi:10.1371/journal.pbio.3001284)

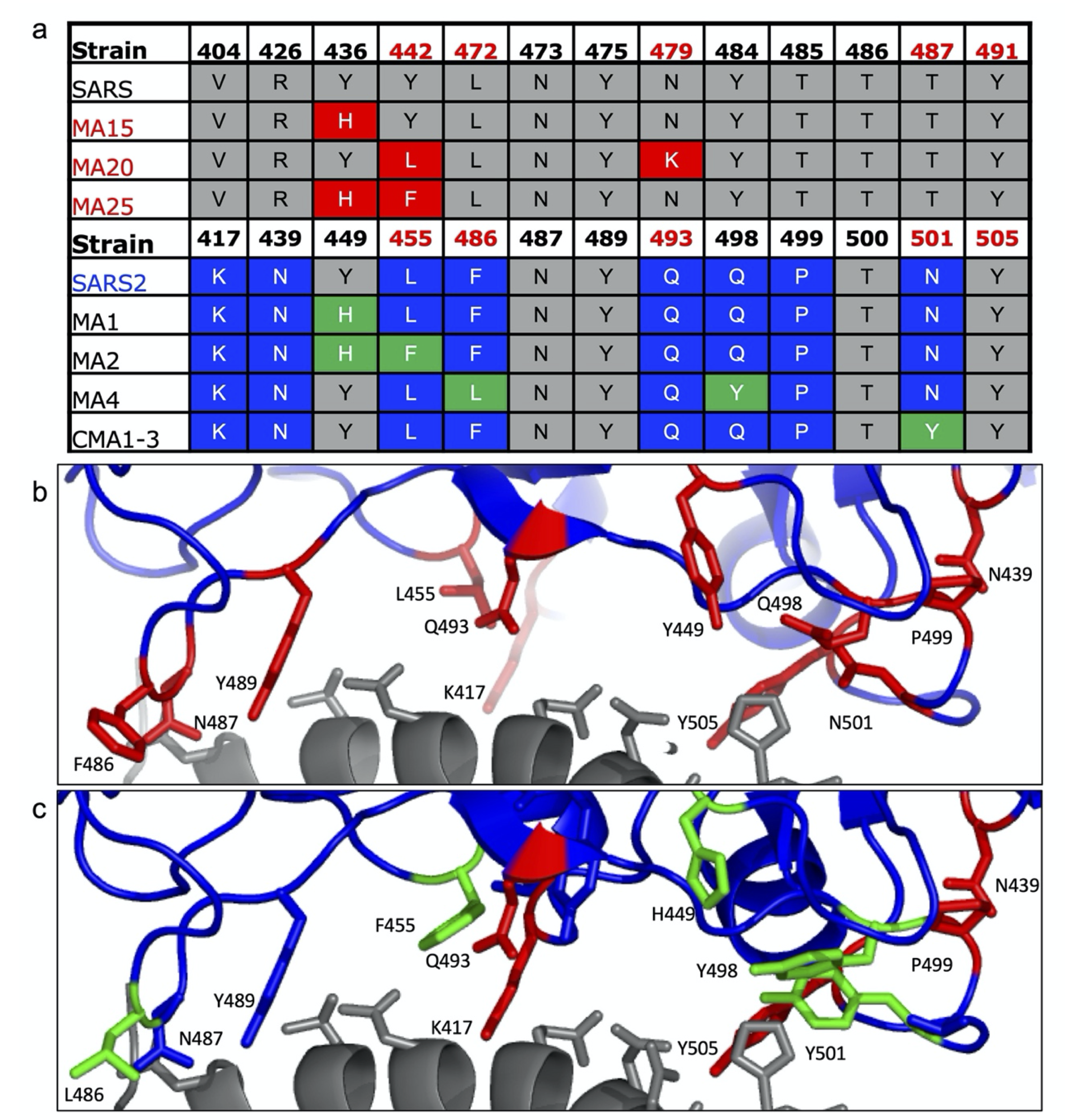

Supplement: S1 Fig — (a) Key amino acid residues found in the RBD of mouse-adapted strains of SARS-CoV were aligned to SARS-CoV-2 and used to design mouse-adapted mutations [13]. Key interaction sites between SARS-CoV spike and ACE2 molecules highlight in red [49]. (b and c) Modeling of key RBD residue interactions with mouse ACE2 (PDB:2AJF) comparing (b) WT SARS-Cov-2 residues versus (c) mutations (green) predicted to improve binding. ACE2, angiotensin converting enzyme 2; RBD, receptor-binding domain; SARS-CoV, Severe Acute Respiratory Syndrome Coronavirus; SARS-CoV-2, Severe Acute Respiratory Syndrome Coronavirus 2; WT, wild-type. (TIFF) [file pbio.3001284.s001.tiff]

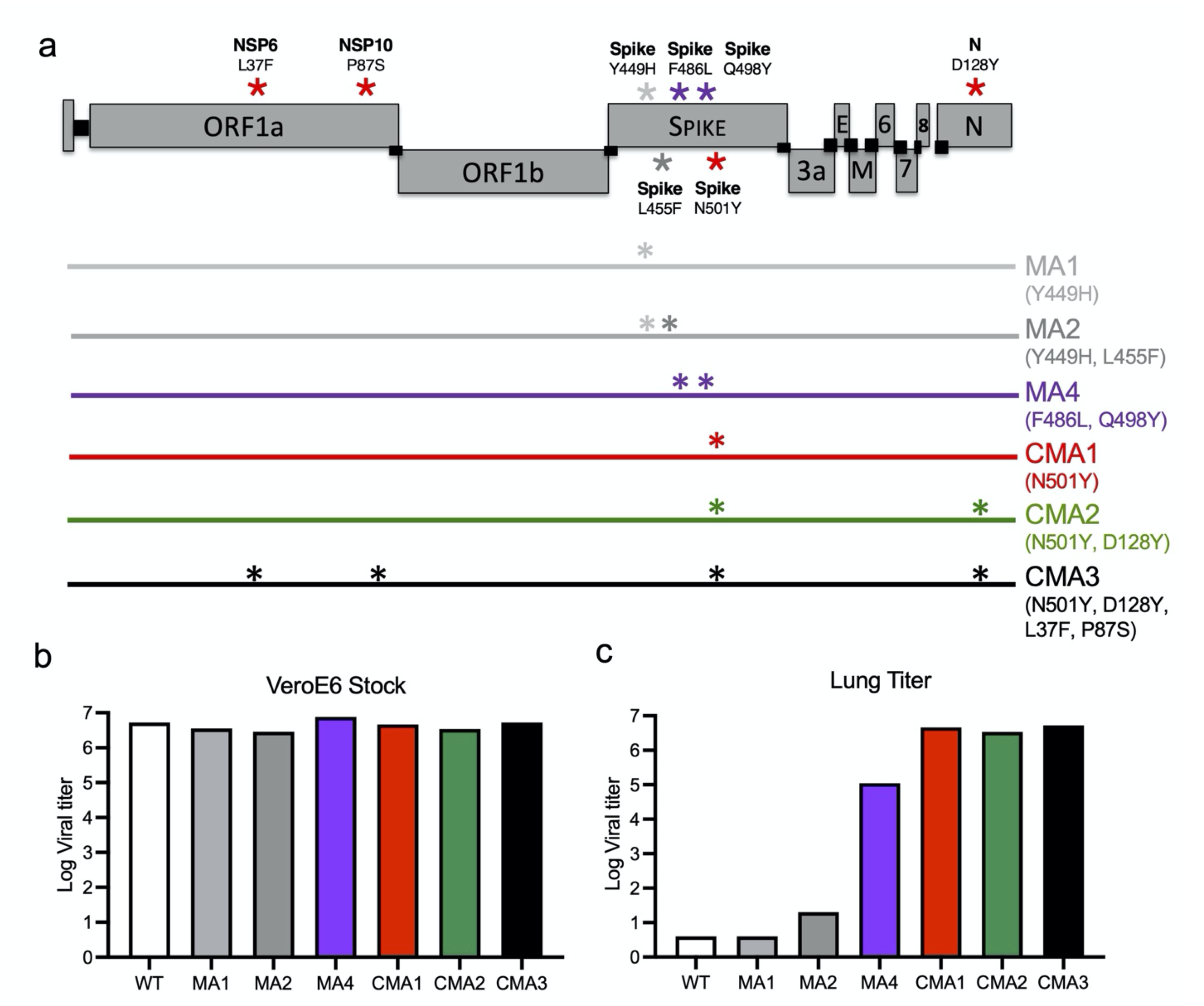

Supplement: S2 Fig — (a) SARS-CoV-2 genome schematic indicating location of amino acid mutations for MA1, MA2, MA4, CMA1, CMA2, and CMA3. (b) Viral replication of stock viruses of MA1, MA2, MA4, and CMA1-3 grown on VeroE6 cells. (c) Viral replication of MA1, MA2, MA4, and CMA1-3 from lung homogenates isolated from infected mice 2 days postinfection (n = 1). Raw data are available in S5 Data. SARS-CoV-2, Severe Acute Respiratory Syndrome Coronavirus 2. (TIFF) [file pbio.3001284.s002.tiff]

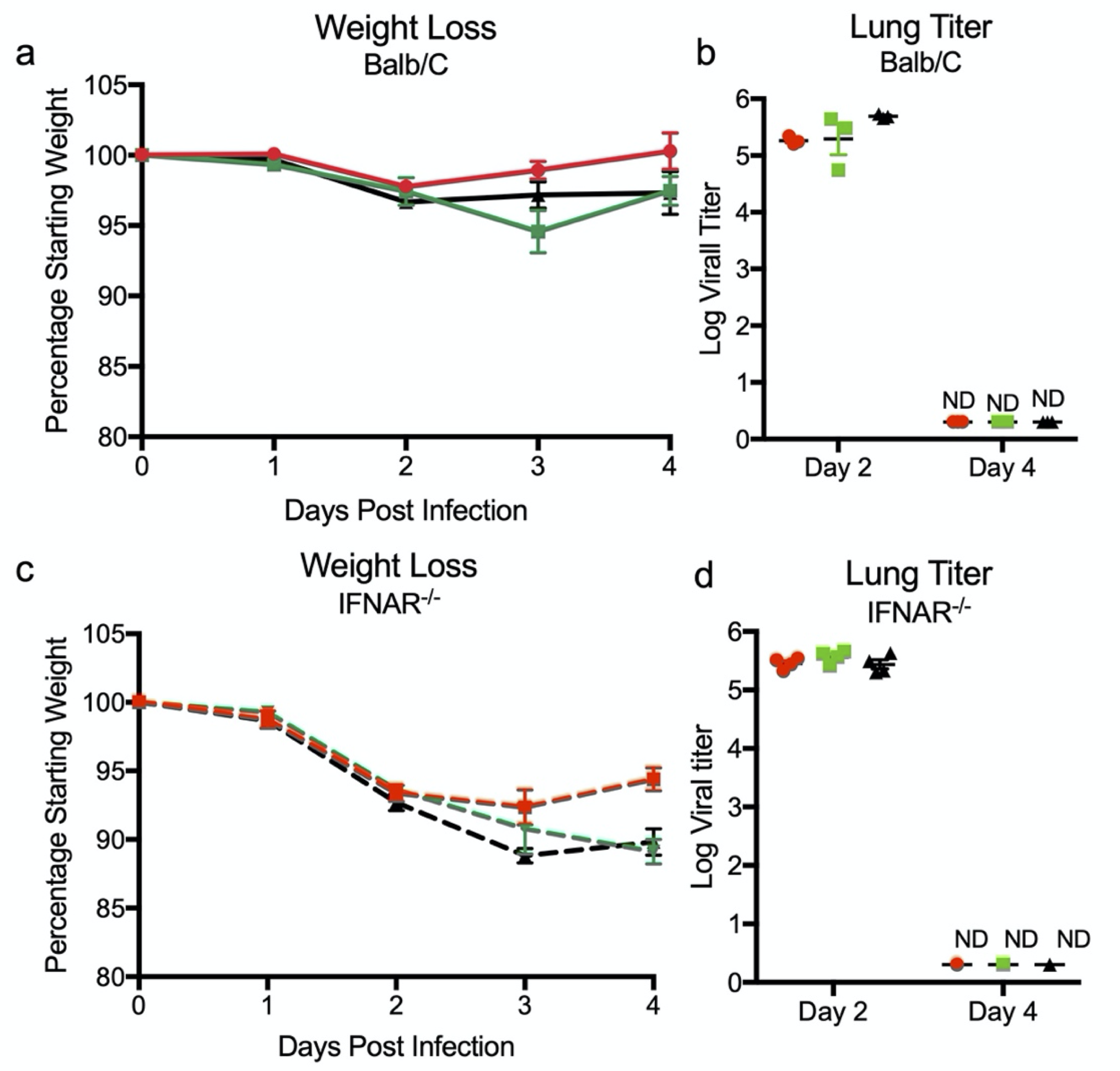

Supplement: S3 Fig — (a and b) Ten-week-old female BALB/c mice infected with 105 PFU of CMA1 (red), CMA2 (green), or CMA3 (black) were examined for (a) weight loss (n = 6) and (b) viral lung titer following infection at days 2 and 4 (n = 3). (c and d) Ten- to 12-week-old female IFNAR−/− SVJ129 mice infected 105 PFU of CMA1 (red), CMA2 (green), or CMA3 (black) were examined for (c) weight loss (n = 6) and (d) viral lung titer following infection at days 2 and 4 (n = 4). Data presented as mean values +/− SEM in (a and c). Raw data are available in S6 Data. ND, nondetected; PFU, plaque-forming unit; SARS-CoV-2, Severe Acute Respiratory Syndrome Coronavirus 2. (TIFF) [file pbio.3001284.s003.tiff]

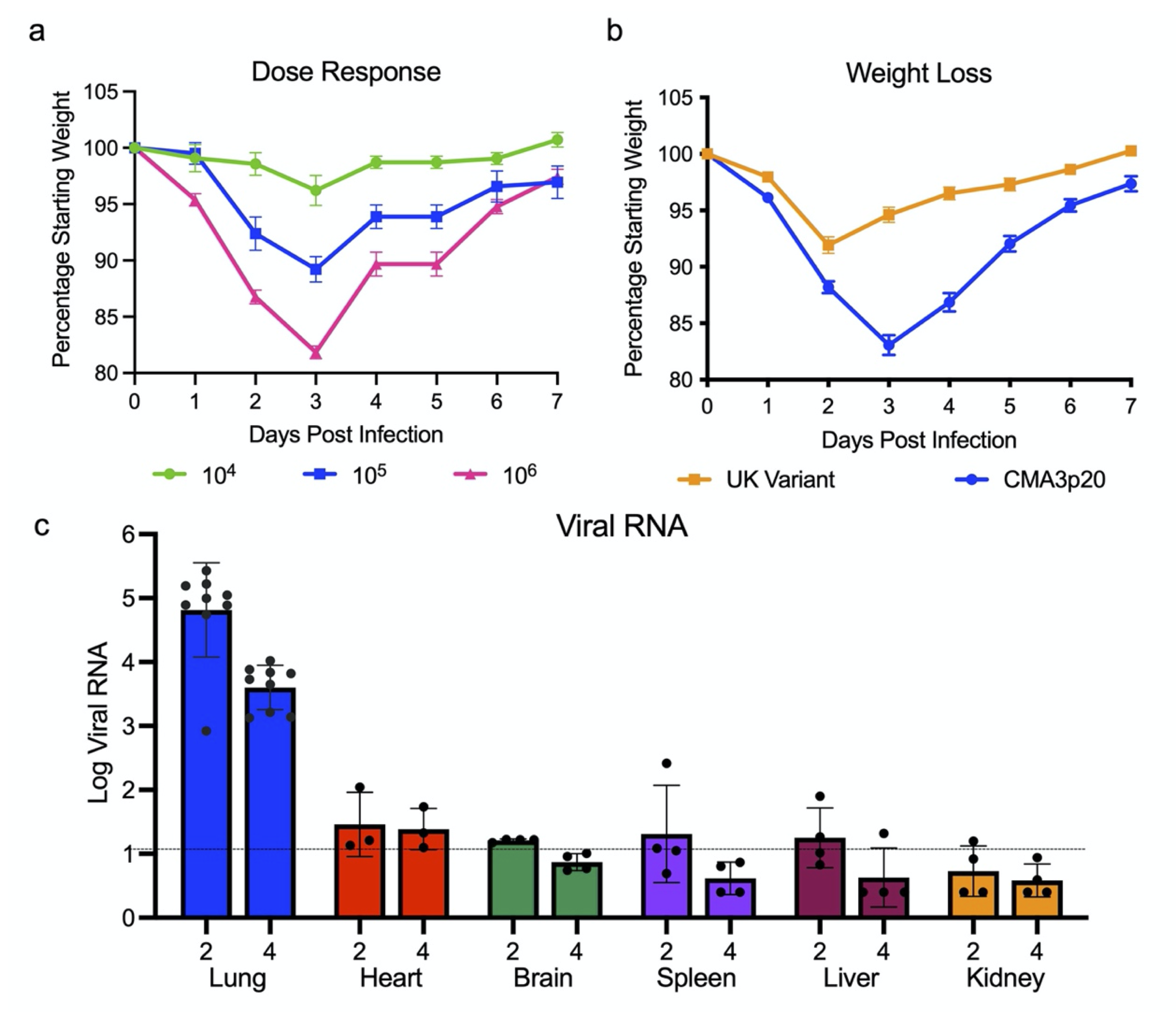

Supplement: S4 Fig — (a) Examination of 10-week-old female BALB/c mice infected with SARS-CoV-2 CMA3p20 at 104, 105, and106 PFU (n = 5). (b) Comparison of weight loss in 10-week old female BALB/c mice infected with 106 PFU of SARS-CoV-2 CMA3p20 (blue) or SARS-CoV-2 variant B.1.1.7 (orange). (c) RT-PCR of viral RNA load found in lung, heart, brain, spleen, liver, and kidney following 105 PFU infection of SARS-CoV-2 CMA3p20 2- and 4-days postinfection. Dotted line signifies viral RNA value derived from mock infected samples. Data presented as mean values +/− SEM in (a–c). Raw data are available in S7 Data. PFU, plaque-forming unit; RT-PCR, real time PCR; SARS-CoV-2, Severe Acute Respiratory Syndrome Coronavirus 2. (TIFF) [file pbio.3001284.s004.tiff]

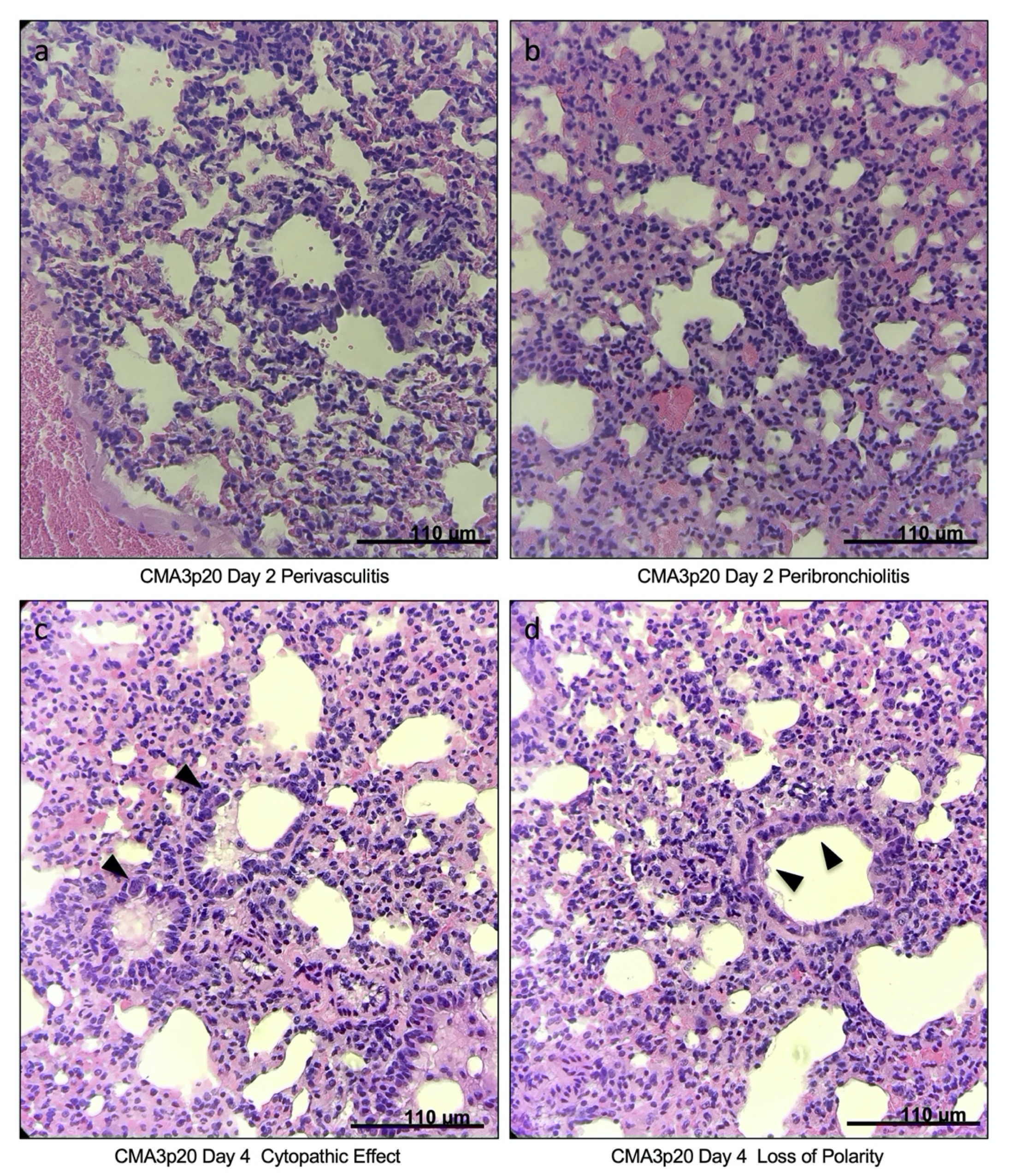

Supplement: S5 Fig — (a–c) CMA3p20-infected animals 2 days postinfection showing (a) perivasculitis and (b) peribronchiolitis. (c and d) CMA3p20 induced lung inflammation and damage 4 days postinfection including (c) cytopathic effect of the virus and (d) loss of cellular polarity as indicated by the black arrows. Magnification at 10× for (a–d). SARS-CoV-2, Severe Acute Respiratory Syndrome Coronavirus 2. (TIFF) [file pbio.3001284.s005.tiff]
